# Oesophageal cancer mortality in Spain: a spatial analysis

**DOI:** 10.1186/1471-2407-7-3

**Published:** 2007-01-03

**Authors:** Nuria Aragonés, Rebeca Ramis, Marina Pollán, Beatriz Pérez-Gómez, Diana Gómez-Barroso, Virginia Lope, Elena Isabel Boldo, Javier García-Pérez, Gonzalo López-Abente

**Affiliations:** 1 Environmental and Cancer Epidemiology Unit, National Centre for Epidemiology, Carlos III Institute of Health. Madrid, Spain

## Abstract

**Background:**

Oesophageal carcinoma is one of the most common cancers worldwide. Its incidence and mortality rates show a wide geographical variation at a world and regional level. Geographic mapping of age-standardized, cause-specific death rates at a municipal level could be a helpful and powerful tool for providing clues leading to a better understanding of its aetiology.

**Methods:**

This study sought to describe the geographic distribution of oesophageal cancer mortality for Spain's 8077 towns, using the autoregressive spatial model proposed by Besag, York and Mollié. Maps were plotted, depicting standardised mortality ratios, smoothed relative risk (RR) estimates, and the spatial pattern of the posterior probability of RR being greater than 1.

**Results:**

Important differences associated with area of residence were observed in risk of dying from oesophageal cancer in Spain during the study period (1989–1998). Among men, excess risk appeared across the north of the country, along a band spanning the length of the Cantabrian coastline, Navarre, the north of Castile & León and the north-west of La Rioja. Excess risk was likewise observed in the provinces of Cadiz and part of Seville in Andalusia, the islands of Tenerife and Gran Canaria, and some towns in the Barcelona and Gerona areas. Among women, there was a noteworthy absence of risk along the mid-section of the Cantabrian seaboard, and increases in mortality, not observed for men, in the west of Extremadura and south-east of Andalusia.

**Conclusion:**

These major gender- and area-related geographical differences in risk would seem to reflect differences in the prevalence of some well-established and modifiable risk factors, including smoking, alcohol consumption, obesity and diet. In addition, excess risks were in evidence for both sexes in some areas, possibly suggesting the implication of certain local environmental or socio-cultural factors. From a public health standpoint, small-area studies could be very useful for identifying locations where epidemiological research and intervention measures ought to receive priority, given the potential for reducing risk in certain places.

## Background

Oesophageal carcinoma is one of the most common cancers worldwide, ranking eighth in terms of incidence and sixth in terms of mortality in 2002 [1]. Its incidence and mortality rates show a wide geographical variation at an international level, with remarkable differences between high- and low-risk areas [1-3]. In Western countries it is a tumour that, on the whole, is relatively infrequent in men and very infrequent in women. However, it is an important problem in economically less developed countries, where the sex ratio is also much closer to unity [1].

Geographic variability is also present at a regional and local level. In Europe, the highest incidence rates among men appear in France and the United Kingdom, where oesophageal cancer is 10 times more frequent than in Greece and Macedonia [2]. Among women, the highest rates are registered by the United Kingdom and Ireland, while the lowest rates are recorded for Mediterranean countries such as Greece and Spain. The most recent incidence data furnished by Spanish tumour registries indicate that European-population-adjusted rates range from 11.51 per 100,000 population in the Canary Islands to 4.45 in Cuenca for men, and from 1.37 in the Canary Islands to 0.55 in Albacete for women [4]. Mortality rates are close to those for incidence, due to the low survival for this tumour (13% and 23% at 5 years in men and women respectively) [5].

The main histopathologic types of oesophageal cancer are squamous cell carcinoma (SCC) and adenocarcinoma (AC). The dominant histological type worldwide is SCC, tobacco and alcohol being the main agents involved in its aetiology [6-8]. For oesophageal AC, the principal risk factors described are gastro-oesophageal reflux and obesity [9-13]. Recent decades have witnessed a surprising increase in the incidence of oesophageal adenocarcinomas in the United States and Europe [14-16], and, though this trend might be partially explained by improvement in the accuracy of histological diagnosis, some authors insist that we are facing a real increase in disease burden [17,18]. SCC, in contrast, registered a relatively stable or declining trend in Western countries over the same time period [14,19]. While the SCC mortality trend could be linked to the decrease in tobacco use in the West, obesity, gastro-oesophageal reflux disease and, possibly, reductions in *Helicobacter pylori *prevalence have all been suggested as contributors to the upward trends in oesophageal AC rates [20].

Despite the fact that an increased risk of oesophageal cancer has also been described in the offspring of affected parents [21], the strong temporal and geographical differences in incidence and mortality at a world and regional level suggest the importance of environmental factors in its aetiology [22]. In Spain, a previous study highlighted the existence of important geographical differences in mortality due to this tumour [23]. Geographic mapping of age-standardized, cause-specific death rates using a greater level of disaggregation could be a helpful and powerful tool for providing clues leading to a better understanding of its aetiology. Hence, this study set out to analyse the spatial distribution of oesophageal cancer mortality at a municipal level in Spain, with the aim of highlighting interprovincial or district patterns.

## Methods

Individual mortality data were used to ascertain the number of oesophageal cancer deaths (International Classification of Diseases, 9^th ^revision [ICD-9], code 150) for the period 1989–1998. These data, which include information on town or city of residence at death, were supplied by the National Statistics Institute for the production of a municipal cancer mortality atlas, after its institutional review board approved the study. The municipal populations, broken down by age group (18 groups) and sex, were drawn from the 1991 census and 1996 municipal roll. These years correspond to the midway points of the two quinquennia that comprise the study period (1989–1993 and 1994–1998). The person-years for each five-year period were estimated by multiplying these populations by 5.

Standardised mortality ratios (SMR) were calculated as the ratio between observed and expected deaths. For the calculation of expected cases, the overall Spanish mortality rates for the above two 5-year periods were applied to each town's person-years by age group, sex and quinquennium.

To draw up these maps, smoothed municipal relative risks (RRs) were calculated using the autoregressive conditional model initially proposed by Clayton and Kaldor [24] and developed by Besag, York and Mollié [25]. Such models are based on fitting spatial Poisson models with two random-effects terms that take the following into account: a) the effects which vary in a structured manner in space (municipal contiguity); and, b) a component that models the effects which vary among municipalities in an unstructured manner (municipal heterogeneity). The model takes the following form

*O*_*i *_~ *Po*(*E*_*i*_λ_*i*_)

log(λ_*i*_) = α + *h*_*i *_+ *b*_*i*_

where: λ_*i *_is the relative risk in area i; *O*_*i *_is the number of deaths in area i; *E*_*i *_are the expected number of cases; *α *is the intercept; *h*_*i *_is the municipal heterogeneity term; and *b*_*i *_is the spatial term.

The models were fitted using bayesian Markov chain Monte Carlo simulation methods with improper priors [26,27]. In Bayesian modelling, prior distributions have to be specified for all unknown parameters even when there is no external information available. Priors, and distributions in general, are improper when they do not integrate to 1. In most cases, improper priors can be used in Bayesian analysis without major problems given that a proper posterior distribution can be obtained [27]. Posterior distributions of RR were obtained using WinBugs [28]. The criterion of contiguity used was municipal adjacency.

It was possible to compile and ascertain the posterior distribution of relative risk on the basis of a single Bayesian spatial model covering all of Spain's 8077 municipal areas. Convergence of the simulations was verified using the BOA (Bayesian Output Analysis) R programme library [29]. Given the great number of parameters of the models, the convergence analysis was performed on a randomly selected sample of 10 towns and cities, taking strata defined by municipal size. Convergence of the estimators was achieved before 100,000 iterations. In the present study, a "burn-in" (iterations discarded to ensure convergence) of 300,000 iterations was performed, and the posterior distribution was derived using 5,000 iterations. The CPU time on a Pentium 2 GHz was 18 hours.

A Geographic Information System (GIS) was used to create municipal maps of SMRs, smoothed RR estimates and the posterior probability that RR>1.

## Results

From 1989 to 1998, a total of 17618 oesophageal cancer deaths were registered in Spain (15274 in men and 2344 in women). Table 1 displays basic descriptive statistics for the population and disease data on both sexes.

**Table 1:** Summary of population and oesophageal cancer mortality in Spain's 8077 towns and cities (calendar period 1989–1998).

	**Total**	**Mean**	**Standard Deviation**	**Median**	**Minimum**	**Maximum**	**No. with zero counts**
**BOTH SEXES**							
Population	39416431	4908.85	42430.43	586	5	2866850	0
Observed	17618	2.18	19.69	0	0	1229	5029
Expected	17579.49	2.17	19.79	0.41	0.00	1346.58	0
SMR		0.77	1.74	0.00	0.00	37.02	5029
RR		0.89	0.23	0.85	0.22	2.29	0
**MEN**							
Population	19212699	2379	20437.56	202	2	1377255	0
Observed	15274	1.89	17.02	0	0	1076	5218
Expected	14093.99	1.75	11.09	0.36	0.00	702.75	0
SMR	-	0.77	1.87	0.00	0.00	40.00	5218
RR	-	0.90	0.29	0.85	0.24	2.17	0
**WOMEN**							
Population	20203732	2501	22827.22	295	1	1561416	0
Observed	2344	0.29	2.77	0	0	153	7088
Expected	2365.66	0.29	2.98	0.05	0.00	207.32	0
SMR	-	0.81	4.96	0.00	0.00	166.67	7088
RR	-	0.86	0.28	0.78	0.00	3.98	7

**Figure 1:**
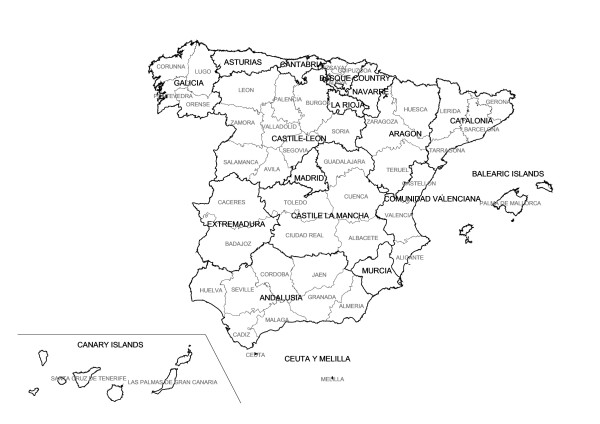
Spanish autonomous regions and provinces.

**Figure 2:**
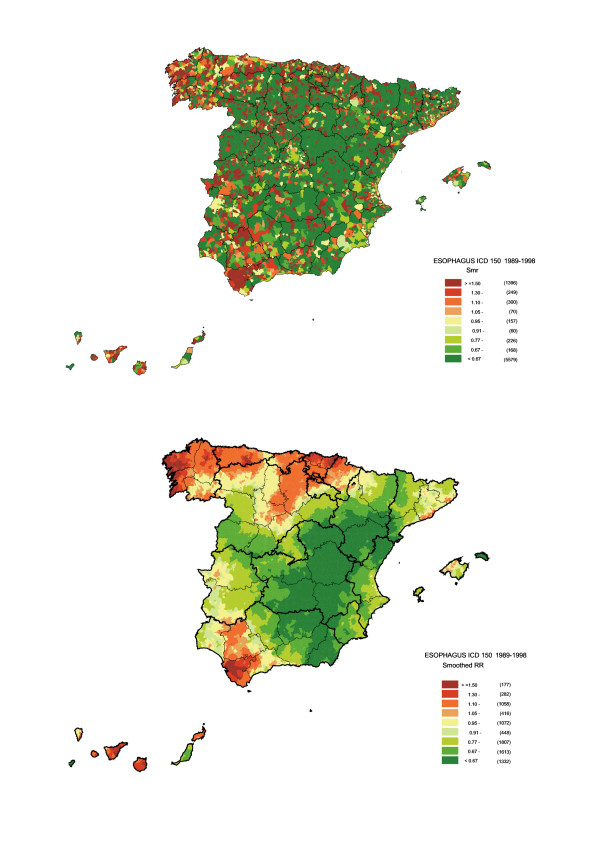
Municipal distribution of oesophageal cancer mortality in Spain (both sexes). Distribution pattern of standardised mortality ratios (SMR) and smoothed relative risk (RR) under the BYM model. Spain 1989–1998.

By way of reference, Figure 1 shows the situation of Spain's autonomous regions and provinces. Figure 2 depicts the distribution pattern of: a) SMR; and b) smoothed RR (both sexes). The SMR map mainly shows towns with and without cases. Despite the presence of a greater number of towns with high risks in some areas of the province of Cadiz, Seville or the provinces along the Cantabrian seaboard (Bay of Biscay), in this map no clear pattern is discernable in the distribution of risk. The smoothed risk map, on the other hand, enables homogeneous areas to be more clearly delimited, showing three areas with excess risk, namely: one in the south of mainland Spain, in the provinces of Cadiz and Seville; one in the north-west of the country, which basically encompasses the coastal provinces of Galicia, Asturias and northern León; and another to the north, covering Cantabria, the Basque Country, the north of Navarre and Burgos, stretching down as far as La Rioja, part of Palencia, Valladolid and even Segovia. Outside mainland Spain, a clear excess risk was likewise in evidence in the Canary Islands. Lastly, some towns in areas around Barcelona and Gerona also registered an excess risk, albeit more moderate.

**Figure 3:**
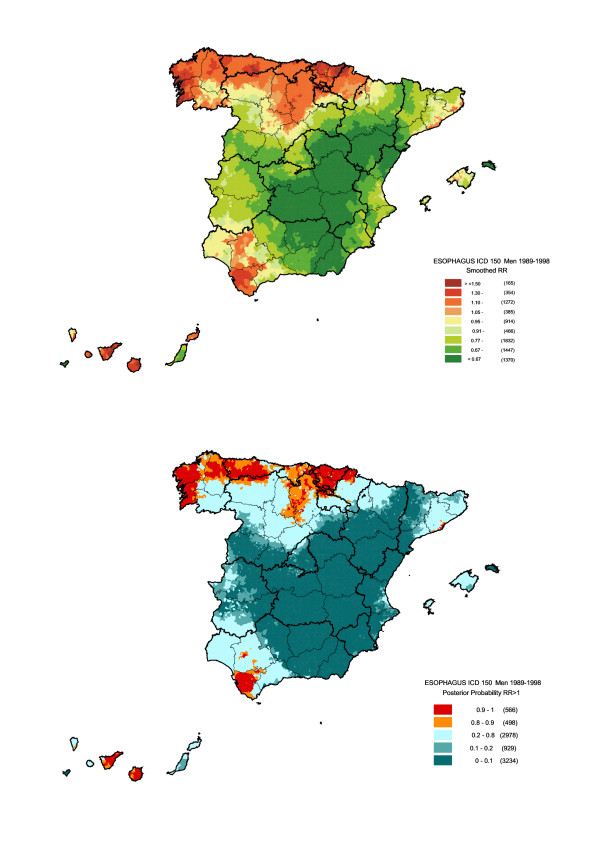
Municipal distribution of oesophageal cancer mortality in men: a) smoothed relative risk (RR); b) posterior probability of RR being greater than 1. Spain 1989–1998.

**Figure 4:**
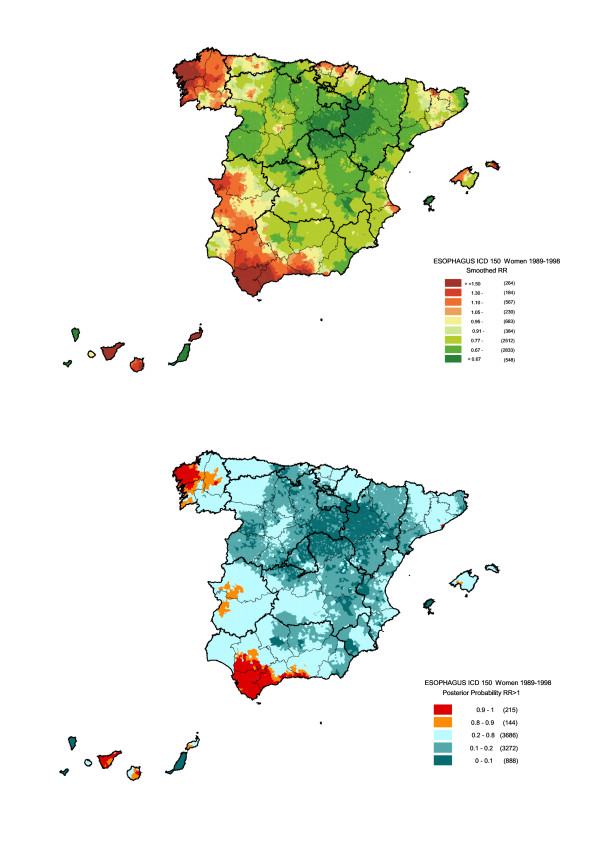
Municipal distribution of oesophageal cancer mortality in women: a) smoothed relative risk (RR); b) posterior probability of RR being greater than 1. Spain 1989–1998.

Figures 3 and 4 depict smoothed relative risk by sex and the likelihood of the estimated RR being greater than 1. As will be seen, the pattern plotted for men determined the combined pattern, since the number of deaths among men was 6-fold that of women. However, the contrast between the maps for men and women enabled differences in the distribution of risk to be observed. Among males, the two areas in the north of Spain that had registered excess risk for both sexes, merged and expanded to include the Cantabrian coastline, from Galicia to Navarre, and the north of the provinces of León, Palencia and Burgos, whereas in women the increased risk in these regions was far more geographically defined, with statistically significant excess risks appearing mainly in the Galician provinces of La Coruña (Corunna), Pontevedra and Lugo. In the south the opposite effect took place, however, with the excess risk observed for men in Andalusia in the provinces of Cadiz and part of Seville, spreading to cover a wider area in women, which included the western area of the province of Malaga, the coast of Malaga and Granada, and the west of Extremadura. A noteworthy feature in men and women alike was the excess mortality in the Canary Islands, which proved statistically significant in towns on the two largest islands, Tenerife and Gran Canaria. Finally, statistically significant RR were also in evidence in the City of Barcelona for both sexes, and in towns along the Barcelona coast for men.

Tables 2 and 3 show a list of towns and cities with 10 or more deaths among men and 5 or more deaths among women, an excess risk of over 1.5, and a posterior probability of over 0.90 that RR would be higher than 1. These arbitrary selection criteria were used in order to select a reasonable number of municipalities with a high and statistically significant risk excess. In line with the findings observed in the maps, towns and cities with risks of over 1.5 were mainly situated: in the case of men, in Corunna, Pontevedra, Guipúzcoa, Vizcaya, Asturias and the Canary Islands; and in the case of women, in Corunna, Lugo, Vizcaya, Cadiz, Malaga and the Canary Islands.

**Table 2:** Municipalities with 10 or more observed cases of oesophageal cancer in men, RRs of over 1.50, and a posterior probability >= 0.90 of having an RR greater than 1. Towns sorted by autonomous region and province. Spain, 1989–1998

**Autonomous region**	**Province**	Town	**Observed**	**Expected**	**SMR**	**RR**	**pp(RR>1)**
Andalusia	Cadiz	Cadiz	75	48.90	1.53	1.51	1.00
Asturias	Oviedo	Aller	26	8.06	3.22	1.63	1.00
		Mieres del Camino	47	23.05	2.04	1.58	1.00
Canary Islands	Palmas	Arucas	17	9.69	1.76	1.50	0.99
		Ingenio	17	5.83	2.92	1.94	1.00
		Telde	37	20.59	1.80	1.70	1.00
	Sta.Cruz de Tenerife	Guimar	14	5.27	2.65	1.86	1.00
		Los Realejos	16	9.10	1.76	1.63	1.00
		Puerto de la Cruz	14	8.41	1.67	1.60	0.99
		Tacoronte	19	5.77	3.29	2.13	1.00
Galicia	Corunna	Arteixo	14	6.76	2.07	1.56	0.99
		Rianxo	12	5.11	2.35	1.72	1.00
		Ribeira	22	9.25	2.38	2.05	1.00
	Pontevedra	Cangas	13	7.71	1.69	1.66	0.99
		Marín	14	7.78	1.80	1.63	1.00
		Moaña	14	5.80	2.41	1.77	1.00
		Mos	11	4.53	2.43	1.57	1.00
		O Porriño	11	4.51	2.44	1.60	1.00
		Vilagarcia de Arousa	16	11.34	1.41	1.54	1.00
		Vilanova de Arousa	15	5.52	2.72	1.99	1.00
La Rioja	Logroño	Haro	11	3.77	2.92	1.50	1.00
Basque Country	Guipúzcoa	Donostia	136	67.42	2.02	1.93	1.00
		Hernani	21	6.88	3.05	2.06	1.00
		Irún	41	19.91	2.06	1.87	1.00
		Lasarte	11	6.08	1.81	1.84	1.00
		Pasaia	16	7.24	2.21	1.93	1.00
		Renteria	26	14.94	1.74	1.81	1.00
		Tolosa	17	7.41	2.29	1.75	1.00
	Vizcaya	Barakaldo	68	42.97	1.58	1.58	1.00
		Bilbao	233	150.99	1.54	1.53	1.00
		Leioa	14	7.55	1.86	1.60	1.00
		Mungia	12	4.40	2.73	1.52	1.00
		Portugalete	31	20.99	1.48	1.58	1.00
		Santurtzi	40	19.02	2.10	1.90	1.00
		Sestao	34	14.33	2.37	1.88	1.00
		Valle de Trápaga	12	4.90	2.45	1.79	1.00

RR = estimated relative risk. SMR = standard mortality ratio. pp = posterior probability that RR>1.

**Table 3:** Municipalities with 5 or more observed cases of oesophageal cancer in women, RRs of over 1.50, and a posterior probability >= 0.90 of having an RR greater than 1. Towns sorted by autonomous region and province. Spain, 1989–1998

**Autonomous region**	**Province**	**Town**	**Observed**	**Expected**	SMR	**RR**	**pp**
Andalusia	Cadiz	Algeciras	9	4.39	2.05	2.10	0.99
		Jerez de la Frontera	23	7.93	2.90	2.24	1.00
		La Línea de la Concepción	9	2.98	3.02	2.77	1.00
		San Fernando	7	3.33	2.10	1.92	0.98
	Malaga	Benamocarra	5	0.13	37.59	3.75	1.00
		Fuengirola	5	1.68	2.99	1.94	0.95
		Ronda	5	1.78	2.80	1.95	1.00
Canary Islands	Palmas	Telde	6	2.46	2.44	1.80	0.97
	Sta. Cruz de Tenerife	La Laguna	8	4.51	1.78	1.73	0.97
		Santa Cruz de Tenerife	17	9.73	1.75	1.75	0.99
Catalonia	Barcelona	Arenys de Mar	5	0.88	5.69	1.56	0.84
Galicia	Corunna	A Coruña (Corunna)	25	15.19	1.65	1.58	0.99
		Coristanco	5	0.65	7.73	2.58	1.00
		Ferrol	12	5.88	2.04	1.76	0.96
		Muros	7	0.84	8.36	3.93	1.00
		Santiago de Compostela	14	5.06	2.77	2.26	1.00
		Teo	6	0.73	8.17	2.37	1.00
	Lugo	Sarriá	6	1.06	5.68	1.74	0.96
Basque Country	Vizcaya	Portugalete	5	2.87	1.74	1.54	0.95
		Santurtzi	6	2.54	2.37	1.80	0.96

RR = estimated relative risk. SMR = standard mortality ratio. pp = posterior probability that RR>1.

## Discussion

This study recorded marked differences in the distribution of oesophageal cancer mortality at a small-area level in Spain and reveals the similarities and differences in the patterns for men and women. Among men, mortality was far greater in the north of the country, though an area with excess was also detected in the south, in the provinces of Cadiz and Seville, as well as in the Canary Islands. Among women, the excess risk in the north of the country was more closely delimited, and there was a noteworthy degree of excess mortality in Galicia and the south-west of the country in particular. These results confirm the existence of some geographical risk patterns partially highlighted in previous studies [23], and suggest the existence of important differences -both geographical and sex-related- in the prevalence of some well-established and modifiable risk factors, including smoking, alcohol consumption, overweight and diet, and to some extent, differences in the distribution of the two main histological types. Furthermore, the fact that in some areas excess risks appear in both sexes suggests the implication of certain local environmental or socio-cultural factors.

Disease maps typically show standardised mortality or morbidity ratios for geographical units [30]. However, the use of SMR for the study of spatial disease patterns in small areas introduces an extra source of variability in the form of random variability, since sparsely populated areas with few or zero cases can generate extreme SMR rates [30]. The Bayesian approach, in contrast, helps identify the underlying geographical pattern. Great part of the variability that appears in the maps before "smoothing" is removed by smoothing, with the result that smoothed RR maps pinpoint high-risk areas. This approach is not free of limitations, however, and there are authors who feel that Bayesian disease-mapping models are essentially conservative [31].

In Spain, mortality represents the only comprehensive and homogeneous source of information on cancer nationwide. Oesophagus is slightly overrepresented in Spanish mortality data, basically due to the inclusion of adjacent gastric cancers [32]. If misclassification were associated with geographical area, then spatial distribution of oesophageal cancer mortality could be affected by this problem. However, death-certificate quality studies undertaken in various regions of Spain do not suggest the existence of important geographical differences in misclassification [32].

Unfortunately, available mortality data do not enable the distribution of the main histological types in Spain to be studied. AC and SCC, though sharing some risk factors, display different spatial distributions and temporal trends worldwide, denoting that they are basically different diseases. Data from existing Spanish tumour registries indicate that incidence of SCC is three to ten times higher than ACC. SCC/ACC ratio do not present a clear geographical pattern, though incidence data are not available for most of the regions with high mortality rates in our country [4].

The implication of tobacco and alcohol in the aetiology of SCC of the oesophagus has been highlighted by a number of studies [6,22,33]. Risk of adenocarcinoma is also increased by tobacco (though the association is weaker than in the case of SCC), but there appears to be little connection with alcohol consumption [6,34]. Furthermore, combined consumption of alcohol and tobacco has a synergic effect that increases risk exponentially, not only for oesophageal cancer, but also for other malignant tumours, such as those of the larynx, oral cavity and pharynx. The fact that in Spain the distribution of male mortality due to oesophageal cancer displays similarities with that of mortality due to the above tumours [23], with risks excesses in south-western Andalusia, in the north of Spain (along the central part of the Cantabrian coastline and the Atlantic coastline of Galicia), as well as in the Canary Islands, points to the importance of these two variables when it comes to explaining the variability encountered in this study among men, given that men drink and smoke more than women [35]. In this respect, risk excesses of dying from bladder and lung cancer have also been registered in south-western Andalusia in men, reflecting the role of tobacco smoking in this pattern [36]. However, the different distribution patterns of these habits [35] are not sufficient to explain the enormous differences found between higher- and lower-risk regions. Studies in high-risk populations have described the implication of certain smoking habit characteristics, such as intensity, duration, or type of tobacco, in the geographical differences in this tumour [37,38], as well as differences in the type of alcoholic beverages consumed locally. In the Canary Islands, moreover, existing tumour registries show the incidence of oesophageal cancer to be the highest in Spain for men and women alike [4], so that it would be of great interest to study which factors might be determining such excess risk in this area.

Gastroesophageal reflux and obesity have been described as the strongest risk factors for oesophageal adenocarcinoma [9-12]. Adenocarcinomas account for around 20% of total oesophageal cancers in some Spanish tumour registries, such as Granada, Mallorca and Murcia, whereas they represent only a 10% of cases in Navarre, Girona and Asturias [4]. Lower exposure to alcohol and tobacco in women could be associated with the greater relative weight of oesophageal adenocarcinoma in Spain. In this respect, the geographic pattern observed for women could be partly associated with differences in the prevalence of obese women, since Extremaduran and Andalusian women register the greatest percentage of obesity in Spain [33]. Galicia and the Canary Islands also have a higher prevalence of obese women than the average level. In the United Kingdom, which has the highest rates of female oesophageal cancer in the EU, high body mass index in early adulthood and low consumption of fruit were associated with increased risk of oesophageal AC in this sex [39], whilst no relationship with alcohol and only a moderate association with smoking were found for SCC [40]. In Scotland, where the highest cumulative rates of oesophageal adenocarcinomas in the world have been reported [3], high rates are observed in both sexes. High intake of saturated fats and high rates of obesity and cigarette smoking have been proposed as factors that could be implicated in these findings [41].

Insofar as diet is concerned, there is consistent evidence to show that consumption of fruit and vegetables reduces the risk of oesophageal cancer (both SCC and AC) [42-47] while higher intake of dietary fat and nutrients found primarily in foods of animal origin and pickled vegetables have been associated with increased risk [44,46,48]. Some evidence of a protective effect of olive oil [45] has been observed, as well as a positive association with butter consumption [45,49]. Furthermore, diet implies exposure to other carcinogens ingested with foods and drinking water, such as *N*-nitroso compounds [47,50,51] or diarrhetic shellfish-poisoning toxins [52]. In France, it has been suggested that differences in risk of oesophageal cancer might be due to exposure to okadaic acid, a tumour promoter that is the main toxin present in French coastal waters. Regular shellfish consumers could be exposed to okadaic acid, also detected in mussels in Galicia, a site of important industrial mussel production [53,54]. It would be interesting to investigate if these marine biotoxins might be linked to the excess risk of oesophageal cancer that appears in this and other geographical areas in our country in subsequent studies.

Similarly connected with dietary habits, consumption of very hot beverages has also been associated with a raised risk of oesophageal cancer in various populations, though its role is not clear [55]. In Europe, the high incidence of squamous cell carcinoma of the oesophagus in the Department of Calvados, the highest risk area in France, was attributed to the local habit of consuming hot alcoholic beverages [49]. With regard to other hot beverages, a recent study has suggested that tea consumption might be the cause of the excess risk of SCC registered by women in the United Kingdom [40]. In South America, the drinking of *mate*, a tea-like infusion, has also been associated with an increased risk of oesophageal SCC [56-58]. In Spain, the role of differences in the consumption of hot beverages in different geographical areas has not been studied.

Social class has also been linked to the occurrence of oesophageal cancer, with a higher incidence being observed in groups having a low socio-economic level [34,59]. In Western countries, low socio-economic level reflects greater exposure to tobacco, alcohol and a worse diet, as well as a greater likelihood of residing in more polluted areas or having little access to the health system. Brown et al [60] estimated that for SCC of the oesophagus the combination of low income, moderate/heavy alcohol consumption, tobacco use, and low daily consumption of raw fruit and vegetables accounted for almost all SCC of the oesophagus in whites (98%) and blacks (99%). They concluded that lifestyle modifications, especially a lower intake of alcoholic beverages, would markedly decrease the incidence of this cancer in both races. This "clustering" of risk factors could be linked to the excess risk detected in Cadiz -where the highest risk of death from cancer in Spain was already described over 15 years ago- ranking it first for the following cancers: lung, oral cavity and pharynx, oesophagus, larynx, and prostate for males; and oesophagus, other uterus, and bone for females [61]. More recently, Benach et al [62] reported that the areas with the highest increased risk of death in both sexes in Spain are clustered in the south-western region of the country, in the provinces of Huelva, Seville and Cadiz. These authors did not elucidate the causes of this clustering of increased mortality but suggested that environmental pollution, occupational exposures and social factors could be involved.

With regard to possible environmental factors which might, at least in part, be determining the pattern detected, the south-west of Andalusia is documented as being exposed to a number of pollutants, including heavy metals and radioactivity in water [63]. In addition, both Galicia and Extremadura are areas with high exposure to naturally produced radioactivity, by reason of the high granite content of their soils [64]. Yet, in Galicia excess risk appears in both sexes, whilst in Extremadura it appears solely in women.

Finally, different infectious agents have likewise been implicated in the aetiology of oesophageal cancer. Whereas infection with Helicobacter pylori, an established carcinogen for gastric cancer, has been associated with reduced risk of oesophageal AC [20], it has also been proposed as a risk factor for oesophageal SCC [65]. Concerning other infectious agents, some studies have suggested that oesophageal human papillomavirus (HPV) infection may be implicated in the development of oesophageal SCC, particularly in high risk areas, in association with known risk factors [66-68] though other studies have found no evidence of a positive association between HPV infection and either form of oesophageal cancer [69]. An association between herpes simplex and Epstein-Barr viruses and oesophageal carcinoma has also been reported in a high-incidence population in China [70]. Though the responsibility of infectious agents in the aetiology of this tumour is yet to be established, it would be an interesting issue to explore in high risk areas in Spain. At present, information of the prevalence of these agents is absent for most Spanish regions.

## Conclusion

The excess risks observed in Galicia, Cadiz and the Canary Islands for both sexes, suggest that the related risk factors could be different to those implicated in the excesses detected in Asturias, Cantabria, the Basque Country, Navarre or the north of Castile & León, which only affect males, and those in Extremadura or the coastal areas of Malaga and Granada, where the excess risks only affect women. Among men, the important differences point, above all, to variations in alcohol and tobacco consumption, including the fact that differing tobacco and alcohol use patterns might be responsible for part of the differences. Among women, differences in the prevalence of obesity, in tandem with different smoking, alcohol use and dietary patterns, might explain, at least in part, the variations in risk linked to geographical area. The possible implication of environmental factors, e.g., drinking water, and infectious agents cannot be ignored. This type of study can serve: to detect areas in which greater stress should be laid on prevention and early detection; and to stimulate further epidemiological study of specific situations.

## List of abbreviations

AC: adenocarcinoma; HPV: human papillomavirus; ICD-9: international classification of diseases, 9 th revision; RR: relative risk; SCC: squamous cell carcinoma; SMR: standardised mortality ratios.

## Competing interests

The author(s) declare that they have no competing interests.

## Authors' contributions

GLA, MP, NA, and BPG were all involved in designing the study. GLA and RR performed the statistical analysis. NA wrote the first draft of the manuscript to which all authors subsequently contributed. All authors made contribution to statistical analyses and interpretation of results, and revised the manuscript for important intellectual content. All authors read and approved the final manuscript.

## Pre-publication history

The pre-publication history for this paper can be accessed here:


